# Evaluating residual stress in additively manufactured nitinol shape memory alloy

**DOI:** 10.1038/s44334-025-00027-y

**Published:** 2025-04-30

**Authors:** Sampreet Rangaswamy, Josiah Cherian Chekotu, Thomas Gillick, Cian Hughes, Jayne Nicholl, David Easton, Caner Şimşir, Dermot Brabazon

**Affiliations:** 1 Advanced Processing Technology Research Centre, School of Mechanical & Manufacturing Engineering, Dublin City University, Dublin, 9 Ireland; 2 I-Form Advanced Manufacturing Research Centre, Dublin City University, Dublin, 9 Ireland; 3 National Manufacturing Institute Scotland, University of Strathclyde, Glasgow, UK; 4 Department of Metallurgical and Materials Engineering, Middle East Technical University, Ankara, Turkey

**Keywords:** Biomedical materials, Actuators, Metals and alloys, Mechanical properties, Characterization and analytical techniques, Mechanical engineering

## Abstract

This study provides a comprehensive analysis of residual stress characteristics in nitinol parts fabricated via laser powder bed fusion (PBF-LB). Unlike previous works that primarily focus on qualitative assessments or single-measurement techniques, this research employs a multi-modal experimental approach, Electronic Speckle Pattern Interferometry-Hole Drilling (ESPI-HD) and X-ray Diffraction (XRD), to achieve a more precise and spatially resolved evaluation of residual stress distribution. Furthermore, the study establishes a direct correlation between residual stress evolution and in situ pyrometric melt pool temperature data, an aspect that has not been extensively explored in prior investigations. A key novel finding is the non-monotonic relationship between volumetric energy density (VED) and residual stress. In this work, laser power was kept constant, and VED was varied by adjusting scanning speed and hatch spacing. The results show that the average residual stress initially increases with decreasing scan speed and hatch spacing, plateaus at a critical threshold, and subsequently decreases. However, residual stress was also found to vary in the build direction, indicating the complex stress distributions and accommodation mechanisms within the material. Additionally, an inverse relationship was recorded between the thermal gradient and VED which challenges conventional assumptions about their relationship. These insights offer a new perspective on optimizing PBF-LB process parameters for enhanced structural performance and long-term reliability of additively manufactured nitinol.

## Introduction

Powder bed fusion using laser beam (PBF-LB) manufacturing technology has gained attention in the metal additive manufacturing sector over the past decade because of its ability to fabricate hard-to-machine metallic (such as Ti alloys) components into a near net shape with little or no postprocessing^1,2^. Nitinol (NiTi) intermetallic alloys are widely used for their shape memory capabilities caused by solid-state diffusionless phase transformations. The phase transformations between the austenite and martensite phases can be thermally induced (shape memory NiTi) or stress-induced (superelastic NiTi) based on the material composition and microstructure for specific applications. By controlling the PBF-LB parameters, these phase transformations can be controlled by altering the mechanical, physical, and thermal properties^3–5^. The PBF-LB of NiTi alloys is at a high developmental pace to produce effective complex structures for a wide range of applications in the medical, energy, and aerospace sectors.

Residual stresses (RS) are often defined as the stresses that remain at equilibrium in a material after certain material processing or treatment steps. These stresses are often exposed when the material is plastically deformed. Since manufacturing processes involve nonuniform loading, RSs are developed and modified at every stage of material processing^6,7^. The RS in PBF-LB originates primarily from process-induced thermal gradients (often referred to as thermal residual stress). The local differences in thermal expansion in a multiphase matrix or between neighboring grains can also cause RS^8,9^. Localized remelting of the powder and fused particles and subsequent rapid cooling (approximately 10^6^ K/s) cause thermal transients, which stimulate stress formation^10^. In PBF-LB, these thermal mechanisms can be described via two models: the temperature gradient mechanism (TGM) and the cool-down mechanism. TGM is linked to stress formation in a single melt track, whereas the latter is linked to stress formation in an entire layer of melted powder^11^.

In NiTi alloys, RS can lead to localized detwinning and plastic dislocations, potentially altering phase transformation characteristics^12^. As a result, the formation of RS in NiTi components plays a critical role in their functional performance. Ma et al. ^13^ reported that increasing the hatch distance in PBF-LB samples resulted in high dislocation densities, leading to local misfits during phase transformation, which correlated with a reduction in the transformation temperatures. Conversely, RS was shown to increase the transformation temperatures, following a Clausius‒Clapeyron stress‒temperature relationship of approximately 8 MPa/K in the work of Liu et al.^14^. This finding indicates that both process-induced RS and high dislocation densities influence the transformation temperatures. Wang et al.^15^ demonstrated that reducing the scanning vector length and employing an appropriate scanning strategy can help minimize RS development in PBF-LB-produced NiTi. In another study by Lu et al.^16^, various martensite morphologies (variants) were attributed to RS in the samples. Additionally, both martensite and intermediate R-phase were observed in the microstructure, potentially resulting from stress accumulation and relaxation during processing. Chekotu et al.^17^ reported that RS from scan overlaps led to microcracks in PBF-LB-fabricated NiTi and recommended substrate preheating to mitigate these stresses. Monu et al^18^. used simulations to model RS in NiTi produced by PBF-LB under different bi-directional scanning patterns. Their predicted stress contours closely matched thermograms from in situ temperature data, providing insights into TGM-type RS formation.

RS can be evaluated through destructive (distortion-based), semi-destructive and nondestructive techniques (diffraction-based). The destructive measurements work on the basis of the strain‒release principle. When an RS-free material is sectioned at a plane, the total summation of stresses normal to the plane should be zero at static equilibrium. If a nonzero strain value is measured normal to the plane, then this difference caused by disturbance of the mechanical equilibrium provides an indication of the RS. The measured strain can then be processed via an analytical or finite element (FE) model to estimate the actual stress values. Common destructive methods include the hole-drilling (HD) method, contour method, and micro-ring core milling analysis^19,20^. A recent modification has been introduced to the traditional hole-drilling method, known as the electronic speckle-pattern interferometry (ESPI)-hole drilling method. ESPI is an innovative, noncontact optical measurement technique designed for the precise measurement of small displacements across the entire field of view. It is a semi-destructive RS measurement technique that is utilized to measure the distribution and magnitude of RS near the surface of a component. It involves drilling a small hole at small increments on the surface of the component, which is illuminated by coherent laser light. The removal of material causes surface displacements and strains as a result of stress release, while the back calculation of these strains is used to determine the original RS at each increment. The method is based on image analysis of images created by the interference of the object beam and the reference beam, where the reference beam is phase shifted and an image is taken at each shift. The ESPI method is therefore more effective in determining the stress relaxations identified by the changes in the speckle pattern. By subtracting this modified speckle pattern from a reference pattern, correlation fringes are obtained, enabling accurate quantification of RS in a highly efficient and noninvasive manner^21,22^.

Diffraction-based methods, such as X-ray diffraction (XRD) and neutron diffraction (ND), rely on two fundamental principles. The first is Bragg’s law presented in Eq. (1), which relates the position of the diffracted peak ($\theta$) to the lattice spacing (${{\rm{d}}}_{\mathrm{hkl}}$) and the wavelength of the incident radiation (${\rm{\lambda }}$). This relationship allows the measurement of the lattice spacing, which can then be compared with the unstrained lattice parameters and ray constants to determine the existing lattice strain^23,24^.

1 $$n\lambda =2{d}_{{hkl}}\sin \theta$$

The second principle is based on Hooke’s law, which relates strain to stress through the material’s modulus of elasticity (${\rm{E}}$). In diffraction-based methods, this relationship is used to estimate stress values. The stress normal to the measured plane is assumed to be zero, allowing the lattice spacing (${\rm{d}})$ to be directly interpreted as the strain measurement. The relationship between the stress (${\sigma }_{\varphi }$), Young’s modulus (${\rm{E}}$), Poisson’s ratio (${\rm{\nu }}$), and slope (${\rm{m}}$) of ${\rm{d}}$ vs. ${\sin }^{2}{\Psi }$ (${\Psi}$ is the angle between the sample surface normal and the diffraction vector) is given by Eq. (2)^25,26^, where ${\rm{\varphi }}$ represents the orientation angle.

2 $${\sigma }_{\varphi }=\left(\frac{E}{1+\nu }\right).\,m$$

The primary objective of this study was to investigate the influence of VED (controlled through variations in scanning speed and hatch spacing while keeping laser power constant) on the development of RS in NiTi samples fabricated via PBF-LB. RS measurements were conducted using two complementary techniques: X-ray diffraction (XRD) for surface-level stress distribution (up to 100 μm depth) and Electronic Speckle Pattern Interferometry-Hole Drilling (ESPI-HD) for stress analysis up to 250 μm in depth. Unlike previous studies that primarily focus on qualitative residual stress assessments or single-measurement methods, this work provides a depth-resolved, multi-modal analysis, offering a more comprehensive understanding of RS distribution in additively manufactured NiTi. Furthermore, the measured residual stresses were systematically correlated with in situ pyrometric infrared (IR) melt pool temperature data—an approach that has received limited attention in the literature to date.

A key novelty of this research lies in its detailed exploration of the relationship between VED, thermal gradients, and RS evolution. While prior studies have acknowledged the role of energy input in stress formation, the findings here reveal a non-monotonic trend in average RS with increasing VED and thermal gradients, alongside depth-dependent variations in stress distribution. Additionally, the inverse relationship between thermal gradients and VED challenges conventional assumptions about their relationship. By elucidating these complex interactions, this study provides new insights into the temperature gradient mechanism of RS formation, demonstrating how precise control of PBF-LB parameters can tailor RS distributions potentially without requiring post-process heat treatments. These findings contribute valuable knowledge for optimizing PBF-LB processing strategies to manage or mitigate RS in NiTi components, ultimately enhancing their structural integrity and functional performance.

## Results and Discussion

### RS analysis based on the XRD and ESPI-HD results

**Fig. 1:**
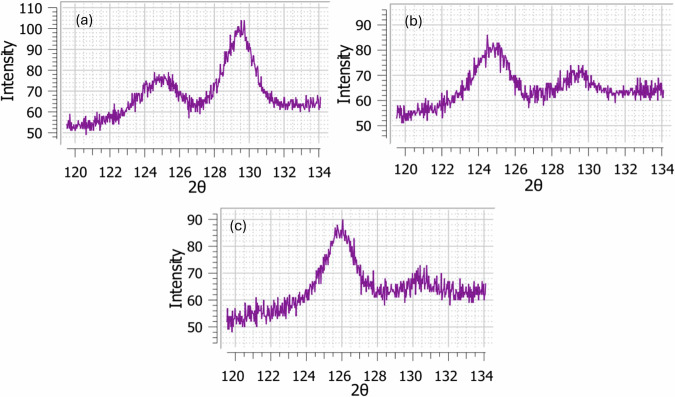
XRD results. Patterns observed for samples (**a**) S1, (**b**) S2 and (**c**) S3.

The XRD and ESPI-HD RS analyses were conducted on the XY plane of the samples (along the z-axis or build direction). In the XRD method, the residual strain (lattice spacing) in the samples was calculated as described earlier through the peak intensities, full width at half-maximum (FWHM) and shifts in the peaks observed. To convert the measured strains into stresses, Eq. (2) was applied using material properties determined from previously conducted in-house experiments, where a Poisson’s ratio of 0.3 and a Young’s modulus of 28 GPa were measured. These values were further validated through published literature^27,28^ to ensure consistency and accuracy. The first stress-measurement trials indicated the presence of a mild-medium texture on the basis of the intensities recorded for 5 positive and 5 negative χ-tilts, which is expected for PBF-LB parts. This resulted in a high relative uncertainty in the results. To improve the results, the number of tilts was increased to 9 positive tilts and 9 negative tilts with a tilt (χ) oscillation of 2°. Moreover, for further improvement, the measurements were carried out on 3 different orientations (φ = 0°, 45°, 90°) with a rotation oscillation of ± 5°. The peak shifts were determined via the cross-correlation method after background removal and absorption correction. The individual stresses for each orientation were determined by fitting an ellipsoid model function to d-sin^2^ψ plots. After this treatment, the relative measurement uncertainties were within 25%, which is considered acceptable for mild-medium textured samples. The XRD patterns of the samples are illustrated in Fig. 1.

The stress components are detailed in Table 1, where ${\upsigma} {\_}{\rm{x}}{\rm{x}}$ and ${\upsigma}{\_}\mathrm{yy}$ represent normal stresses, ${\uptau} {\_}{\rm{x}}{\rm{y}}$ denotes shear stress, and ${\upsigma}{\_}{\rm{Max}}$ and ${\upsigma}{\_}{\mathrm{Min}}$ are principal stresses. To derive an average generalized stress value from the RS measurements, the von-Mises stress was calculated using the normal and shear stress components, as expressed in Eq. (3). The analysis revealed that the stresses exist in both the tensile and compressive regimes. Specifically, compressive stresses are predominant near the surface, whereas tensile stresses become more prevalent deeper within the material. Compressive stresses can be beneficial, as they increase the resistance of a material to crack initiation and improve its fatigue life. In contrast, tensile stresses may increase the susceptibility to crack propagation^29,30^.

3 $${\sigma }_{vonMises}=\sqrt{{(\sigma \_xx)}^{2}+{(\sigma \_yy)}^{2}-(\sigma \_xx\times \sigma \_yy)+3{(\tau \_xy)}^{2}}$$

**Table 1:** Stress components in the XY plane for samples S1, S2, and S3

Sample	Depth [μm]	Method	σ_xx [MPa]	σ_yy [MPa]	τ_xy [MPa]	σ_Max [MPa]	σ_Min [MPa]	Von-Mises [MPa]
S1 [Low VED]	<100	XRD	−11.4	−4.1	7.1	0.3	−15.7	15.85
	100	ESPI-HD	19.7	22.4	0.58	22.5	19.5	21.20
	150	ESPI-HD	3.91	8.07	−0.95	8.28	3.7	7.18
	200	ESPI-HD	11.7	8.53	−1.65	12.4	7.84	10.86
	250	ESPI-HD	15	4.84	10.3	21.4	−1.6	22.23
Average			7.78	7.95	3.08	12.98	2.75	13.77
S2 [Mid VED]	<100	XRD	0.4	−11.6	4.7	2	−13.2	14.34
	100	ESPI-HD	54.1	61.2	−5.64	64.3	51	58.79
	150	ESPI-HD	59.8	58.4	1.06	60.4	57.8	59.14
	200	ESPI-HD	36.4	8.41	2.4	36.6	8.21	33.27
	250	ESPI-HD	15.9	−4.29	−7.66	18.5	−6.87	22.70
Average			33.32	22.42	−1.03	36.36	19.39	37.65
S3 [High VED]	<100	XRD	0.7	7.4	−5.9	10.9	−2.8	12.43
	100	ESPI-HD	−8.97	−0.63	−3.47	0.62	−10.2	10.55
	150	ESPI-HD	35.3	25.4	−11.8	43.1	17.6	37.58
	200	ESPI-HD	35.1	35.7	−2.44	37.9	33	35.66
	250	ESPI-HD	32.9	20.6	−0.29	32.9	20.6	28.80
Average			19.00	17.67	−4.78	25.09	11.64	24.05

### Effect of increasing VED by reduced scanning speed and hatch spacing on RS

**Fig. 2:**
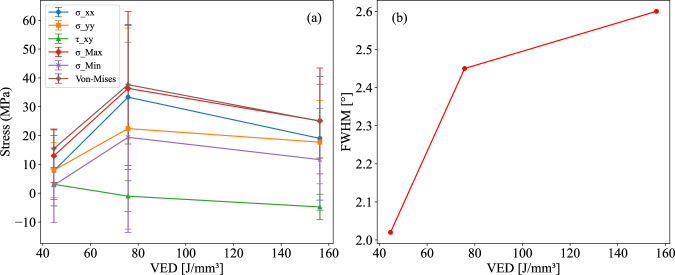
Effect of VED on RS and FWHM. **a** Stress response to increasing VED, illustrating trends in normal stresses, shear stress, principal stresses and von-Mises stress, and (**b**) effect of increasing VED on the FWHM from XRD measurements.

Figure 2a illustrates the relationship between the stress components and VED along with error bars calculated based on standard deviation of the stress values in Table 1. Our work focused on increasing the VED by decreasing the scanning speed and hatch spacing, while keeping the laser power constant. The data show that the RS behavior varies significantly with depth and VED. When examining the stress trends at different depths, distinct variations emerge. For instance, at depths less than 100 μm, the von-Mises stress consistently decreases with increasing VED. At deeper regions, specifically at 200 μm and 250 μm, a consistent increasing trend in von-Mises stress is observed with rising VED. This depth-dependent variation in RS highlights the need for a more nuanced interpretation of VED’s effects across different regions of the sample. Upon analyzing the averaged values of the stress components and von-Mises stress, it can be observed that, as VED increases, RS initially rises and then decreases. This trend suggests that at lower to intermediate VED levels, energy absorption within the material intensifies, likely causing localized heating that leads to uneven expansion and contraction, which in turn generates RS. Additionally, at these lower VED levels, rapid cooling rates can promote the formation of martensitic phases, further contributing to increased RS^31^. As VED continues to increase, the melt pool reaches higher temperatures and expands, resulting in a larger melt pool volume and reduced cooling rates. This mitigates the steep thermal gradients associated with high cooling and solidification rates, which are known to drive detrimental tensile RS. The elevated melt pool temperatures and slower cooling rates promote stress relaxation, as the material has more time to accommodate thermal strains during solidification. Consequently, the reduction in the RS at a higher VED is observed as the cooling rate decreases. Since scanning speed affects solidification rates, it is likely that both factors—higher thermal energy input and slower solidification—contribute to the stress relaxation observed at high VED. Materials exposed to high energy densities can adapt more effectively to thermal changes, leading to lower RS^32^. Furthermore, phase transformations, such as those from martensite to austenite at elevated temperatures, induce volume changes that aid in redistributing internal stresses, contributing to an overall reduction in RS levels^33,34^. Mugwagwa et al.^35^ also reported that intermediate VED resulted in higher RS than both low and high VED in PBF-LB, similar to the findings of this study. These authors attributed the higher porosity in the low- and high-VED samples as the cause for lower RS. The porosity related to VED in PBF-LB can arise from two main causes. Excessively high VED can result in overheating, leading to keyhole porosity, whereas insufficient VED can cause underheating, leading to a lack of fusion porosity. However, in addition to VED, variations in scanning speed impact solidification dynamics, further influencing both RS and defect formation. Therefore, while VED remains a key parameter in RS development, differences in solidification rates resulting from varying scanning speeds must also be considered when interpreting the observed trends in the sample stress profile.

Figure 2b shows the impact of increasing the VED on the FWHM of the diffraction peaks, as measured via XRD. A sharp increase in the FWHM is observed when the VED increases from low to intermediate levels, followed by a less significant increase from intermediate to high levels. As indicated by the Scherrer equation, the FWHM is directly related to strain within the material and inversely related to crystallite size: larger FWHM values generally signify smaller crystallites, whereas smaller FWHM values are associated with larger grains and better crystalline quality^36^. With increasing VED, the elevated energy input promotes atomic mobility during solidification, lowers the cooling rate, and consequently leads to grain growth. According to the Hall-Petch relationship, a reduced grain size increases the material strength. However, the greater grain boundary area in smaller grains limits dislocation movement, which in turn increases internal stress levels^37,38^. In our study, the grain size reduction is more pronounced from low to intermediate VED and less so from intermediate to high VED, as evidenced by the changes in the FWHM. This trend aligns with the observed RS, where the intermediate VED results in the highest RS, which is likely due to the rapid decrease in the grain size at this level. In contrast, the reduction in RS at high VED may result from a less steep overall grain size reduction, potentially due to the formation of larger grains. Lin et al.^39^ previously reported this unique grain size behavior in NiTi processed via PBF-LB, noting that the grain size can vary nonlinearly with VED, increasing with increasing VED in some cases and decreasing in others. Additionally, elevated VED levels during PBF-LB of NiTi have been shown to increase melt pool temperatures, which can lead to Ni evaporation and the formation of fine martensitic grains and precipitates such as Ti_2_Ni and Ni_4_Ti_3_^40,41^.

### Thermal data analysis for the correlation between the RS, VED and thermal gradient

**Fig. 3:**
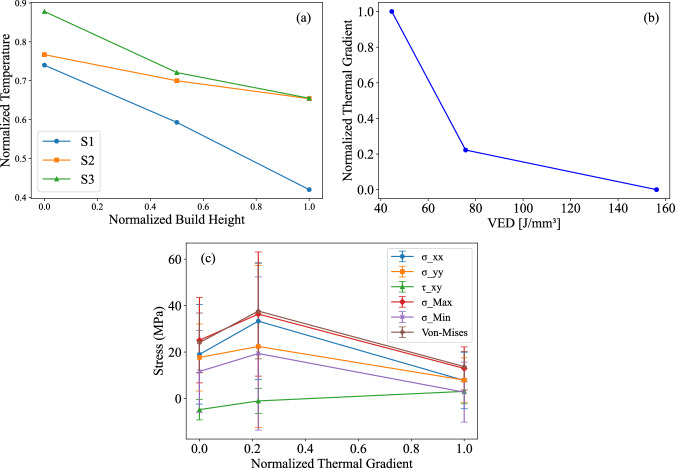
Correlation between build height, layer temperature, VED, TG and RS. **a** Thermal profiles showing temperature variations with build height for samples S1, S2, and S3, (**b**) correlation between VED and the thermal gradient, and (c) correlation between the thermal gradient and residual stress components.

The IR thermal data collected during the PBF-LB printing process were analyzed across the XY plane for each sample, aligning with the RS measurements. The thermal profiles, which represent layer temperatures as a function of build height (z-axis), are presented in Fig. 3a. The recorded temperature can be seen to decrease with build height. This trend can be attributed to multiple factors. As more layers are deposited, the additional input energy results in a temperature rise locally. This heat conducts toward the substrate to raise the temperature toward the base of the sample. The substrate raises to a certain temperature threshold level before stabilizing. The upper layers experience more heat dissipation to their surrounding environment due to the convective gas flow^42,43^. These factors contribute to the observed temperature gradient with increasing build height. Another trend observed is the progressive temperature increase from samples S1 to S3 with increasing VED, indicating that higher VED levels lead to higher melt pool temperatures. The thermal gradient (TG) for each sample was calculated using Eq. (4), which assesses changes in the normalized temperature with respect to the build height:

4 $$\nabla T(z)=\left|\frac{{\partial}{T}_{norm}}{{\partial}z}\right|$$

The effect of VED (reduced scanning speed and hatch spacing) on the TG is illustrated in Fig. 3b, which shows a decrease in the TG with increasing VED. As VED increases, the larger melt pool volume and lower cooling rates promote a more uniform thermal distribution, leading to lower thermal gradients. Figure 3c shows the relationships between TG and the various stress components previously discussed along with error bars calculated based on standard deviation of the stress values in Table 1. Similar to the VED-RS trend, the average RS is highest at intermediate TG level. Owing to the inverse relationship between VED and TG, these trends mirror one another: a low TG corresponds to high VED, and vice versa. Initially, increasing TG increases RS due to disparities in thermal expansion and contraction. However, with further increases in TG, phase transformations (such as martensite-to-austenite) and increased plastic deformation contribute to reducing RS^44^.

Xue et al.^45^ investigated the effect of increasing VED in PBF-LB by reducing hatch spacing, similar to this study, and observed a reduction in RS, delamination, and cracking. Lin et al.^39^ also reported that a higher VED, achieved by reducing the hatch distance, improved the thermal gradient, reduced the cooling rate, and lowered the RS, resulting in a favorable <001> texture in the PBF-LB of NiTi. However, as indicated by Monu et al. ^46^, the relationship between VED and RS is not always straightforward. Their study showed that an increase in VED resulted in higher thermal gradients and increased RS in PBF-LB NiTi, underscoring the complex nature of RS evolution in this material. This discrepancy suggests that, while adjusting VED does influence RS, its effects are material- and process-dependent. Liu et al.^47^ observed a consistent increase in RS in the PBF-LB of NiTi when they increased both laser power and scanning speed while keeping hatch spacing and VED constant. This finding suggests that laser power plays a critical role in driving RS, highlighting the need for a more nuanced approach in optimizing process parameters. Furthermore, Li et al.^48^ reported a trend similar to ours in their study on RS-induced cracking, where RS first increased with VED and then decreased with further increases in VED.

More broadly, the effect of VED on RS is highly material-dependent^49^. Additional studies examined this relationship in different materials, specifically steels, and presented trends between RS and VED for these materials^50,51^. Simson et al.^50^ found that RS, that was measured on the top surface of austenitic 316 L stainless steel samples, increased considerably between low to mid VED values and remained relatively constant at higher VED values. Narvan et al.^51^ also examined the relationship between RS and VED on the top surface of H13 tool steel samples. This study found that compressive stresses existed on these surfaces and were observed to increase significantly from low to mid VED values and were then seen to decrease slightly moving to higher VED values. These contrasting results across different materials reinforce the need for continued research into how PBF-LB process parameters influence RS, including for NiTi specifically.

Our study’s results, particularly the observed depth-dependent variation in RS, emphasize the need for further investigation into the interaction between scanning speed, hatch spacing, and laser power in determining RS. Moreover, the stochastic nature of the PBF-LB process and practical constraints on sample characterization limit the number of specimens we can measure. The time-intensive nature of stress measurement techniques, such as XRD and ESPI-HD, restricts the dataset size. Although the results in this study provide valuable insights into the role of VED in RS development, there is much more process window space that could be investigated to provide a more detailed understanding of the relationship between the parameters and RS. Further studies could also focus on varying laser power while keeping scanning speed constant. This would allow for more of an understanding of how power influences thermal gradients and RS, independent of other parameters. Additionally, VED can be calculated based on laser spot size instead of hatch spacing, providing a different perspective on how energy is distributed across the material during processing. Expanding experimental datasets and leveraging in situ monitoring techniques in these ways could help provide a more comprehensive understanding of the complex thermal-mechanical interactions governing residual stress evolution in PBF-LB components.

## Methods

### PBF-LB process

The NiTi powder used was composed of 50 at.% Ni and 50 at.% Ti. The samples were fabricated using Aconity MINI (GmbH) PBF-LB machine equipped with a Nd:YAG fiber laser system from IPG Photonics, which has a wavelength of 1068 nm and a maximum power capacity of 200 W. The build chamber was installed with a rubber-based recoater blade. The chamber was purged with argon gas (99.999%) to ensure a minimum oxygen content ( < 50 ppm) throughout the melting and solidification process. A powder supply factor of 1.8 (nearly twice the layer thickness) was used in the print process to ensure sufficient powder spread. To reduce spatter on fresh layers, the printing sequence started from the inert gas exit side of the build chamber.

Printing was performed using the laser parameter levels shown in Table 2, with a layer thickness of 40 μm and a spot size of 50 μm. The sample dimensions were 20$\times$10$\times$7.5 mm^3^. The layer thickness was determined on the basis of the feedstock particle size distribution to ensure good powder spread and flowability on the build platform. These parameter sets can be used to calculate the volumetric energy density (VED), which can be calculated via Eq. (5):

5 $${VED}=\frac{P}{v\times h\times t}$$

where ${\rm{P}}$ is the laser power (W); ${\rm{v}}$ is the scan speed (mm/s); ${\rm{h}}$ is the hatch spacing (mm); and ${\rm{t}}$ is the layer thickness (mm). The laser power was kept constant, while the scanning speed was decreased in increments of 300 mm/s, and the hatch spacing was reduced by 15 μm steps to achieve increasing VED.

**Table 2:** Parameter levels used and respective volumetric energy densities

Sample	P (W)	v (mm/s)	h (µm)	VED (J/mm^3^)
**S1**	150	1200	70	44.64
**S2**	150	900	55	75.76
**S3**	150	600	40	156.25

The samples were arranged on the substrate plate at an in-plane angle of 30° to reduce any impact load on the re-coater assembly and to allow more gradual gas flow/fume removal over the layers in the chamber during printing. A simple stripe scanning strategy was maintained with a rotation of 90° for each subsequent layer. This prevents any unwanted overheating around the corners of the sample. The build chamber ambient temperature during the process was 20 ± 1 °C.

### Thermal data monitoring

The thermal data of the molten pool were captured using two pyrometers from KLEIBER Infrared GmbH, which were employed in the Aconity PBF-LB machine. These pyrometers detect the light emitted from the area where the laser interacts, which falls within the 1500–1700 nm wavelength range. The resulting infrared (IR) data are recorded in terms of voltage (mV) units and the corresponding coordinates on the build plate (x, y, z). In this study, we generated graphical representations to visualize the temperature variations across different sections of a sample. The rate at which the data were collected was 100000 samples per second. Approximately 6 million data points were collected for a single layer, each comprising both coordinates and temperature values in mV. To depict the thermal profile, the temperature readings were transformed into a normalized scale ranging from 0 to 1.

### Experimental RS measurement

**Fig. 4:**
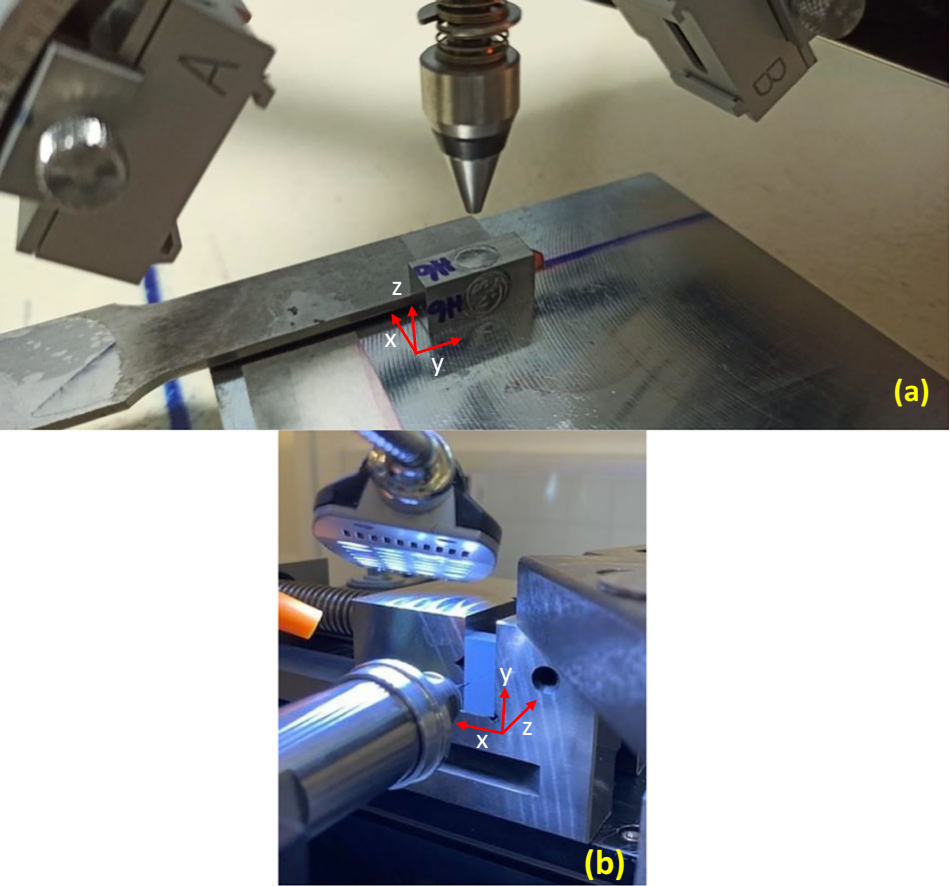
Experimental setup for RS measurements. **a** XRD measurement setup and (**b**) ESPI-hole drilling measurement setup, with the three axes of the sample (x, y, z), where the z-axis represents the build direction.

XRD measurements were performed using a 3-circle portable-type stress diffractometer in modified-χ mode. Considering the fluorescence of copper with nickel and chromium with titanium, it was not possible to measure the stresses with those well-known X-ray sources. As alternatives, Mn‒Kα and Ti‒Kα radiation were used, and the best results were acquired using Ti‒Kα radiation with an average wavelength of 0.2752 nm. Using this radiation, considering the diffraction peaks above 2θ > 120°, it was possible to perform the measurements using the {1 1 0} or {2 0 0} set of planes of the austenite phase. Although a higher diffraction angle is favored for the {2 0 0} set of planes, the peaks were quite broad (reaching 10°–15°) because of the small crystallite size and high dislocation density, which resulted in the extension of the detection range. Thus, the measurements were carried out on a (2 1 1) set of planes located at approximately 134°. The tube voltage was set to 30 kV, and the tube current was 7 mA. Before the measurements, the focal distance was calibrated using both commercially pure Ti (CP-Ti) and Ti-6Al-4V powders, which are certified by the diffractometer manufacturer. In both cases, the focal distance was adjusted so that the total error in the stress calculations was lower than ±10 MPa. Figure 4a shows the XRD measurement setup.

For ESPI-hole drilling measurements, the sample surface was spray painted to reduce reflections. A drilling operation was performed precisely at the center of the sample using a Ø0.79 mm 2 flute carbide TiAlNi-coated inverted end mill. The drilling process involved incremental steps of approximately 50 μm until a depth of 250 μm was reached. A feed rate of 0.025 mm/s and a spindle speed of 30 × 1000/min were selected as the drilling conditions, according to the recommendations of the operation manual. Simultaneously, a laser beam was employed to illuminate the sample, while a camera captured the distinctive speckle pattern generated by the surface roughness. This camera image captures the interference between the object beam and the reference beam. The reference beam underwent phase shifting, and an image was acquired for each phase shift, resulting in a total of four images to characterize the surface conditions before and after each drilling increment. These four images facilitated the computation of a phase angle for every pixel. This phase angle information was subsequently translated into surface displacement. Figure 4b shows the ESPI-hole drilling setup.

## Data Availability

Any additional data apart from what is provided within the manuscript can be supplied upon request.
